# Primary Neoplasms of the Seminal Vesicles: A Narrative Review of Carcinoma and the Rarer Non-Epithelial and Biphasic Tumours

**DOI:** 10.3390/diagnostics16182943

**Published:** 2026-09-11

**Authors:** Athanasios Zachariou, Grigorios Daligkaros, Maria Filiponi

**Affiliations:** 1 Department of Urology, Ioannina University, 45500 Ioannina, Greece; 2 Outpatient Urology Department, Physical Medicine and Rehabilitation Centre Kentavros, 38222 Volos, Greece

**Keywords:** seminal vesicle, carcinoma, adenocarcinoma, seminoma, sarcoma, mixed epithelial and stromal tumour, CA-125, immunohistochemistry

## Abstract

Primary carcinoma of the seminal vesicle is an exceptionally rare genitourinary malignancy, with evidence limited largely to case reports and small retrospective series. Diagnosis requires exclusion of more common secondary involvement from prostate, bladder, or rectal carcinoma through integration of clinical findings, imaging, histomorphology, and immunohistochemistry. Patients typically present with nonspecific symptoms and often have locally advanced disease. Computed tomography identifies retrovesical masses, while magnetic resonance imaging provides superior local staging. Core biopsy may establish the diagnosis, although definitive classification may require resection. The typical immunophenotype is CK7-positive, often with CA-125 expression, and negative for PSA, PAP, and NKX3.1; however, no marker profile is invariable. A contemporary panel including GATA3, p63/p40, uroplakin markers, CK20, p53, CD44, CDX2, and SATB2 is important for excluding urothelial and intestinal primaries. Conventional papillary adenocarcinoma is the predominant histological type, with mucinous, clear cell, squamous, and neuroendocrine variants also reported. Non-carcinomatous primary seminal vesicle neoplasms, including germ-cell tumours, sarcomas, solitary fibrous tumour, and mixed epithelial and stromal tumour, form an important differential diagnosis. Complete surgical excision with negative margins is the treatment most consistently associated with prolonged survival in the reported cases and is generally regarded as the only potentially curative option, whereas radiotherapy, chemotherapy, and hormonal therapy are selectively used for advanced or recurrent disease. International registries and collaborative molecular studies are needed to improve diagnosis, treatment, and follow-up.

## 1. Introduction

The seminal vesicles are a pair of coiled, sacculated tubular glands situated posterolateral to the base of the urinary bladder and superior to the prostate. Embryologically derived from the mesonephric (Wolffian) duct system, they contribute the majority of the volume of the ejaculate. Despite their intimate anatomical relationship with organs that are among the commonest sites of malignancy in men, the seminal vesicles are themselves an exceptionally infrequent site of primary neoplasia [1,2]. The overwhelming majority of tumors identified within the seminal vesicle represent secondary involvement by direct extension from an adjacent prostatic, vesical, or rectal carcinoma, rather than a cancer of seminal vesicle origin [3,4,5,6].

Primary seminal vesicle carcinoma (PSVC) was first reported by Lyons in 1925 [1], and in the century since only about 100 histologically confirmed cases have accumulated in the English-language literature [7,8]. This scarcity has profound consequences. No prospective data exist; the entire evidence base is composed of single case reports, small autopsy series, and narrative reviews [9]. There is no dedicated staging system, no consensus treatment algorithm, and no validated prognostic model. Most practicing urologists and pathologists will never encounter a case, and when they do, the tumor is easily misattributed to a commoner pelvic primary [10,11].

The clinical importance of the entity is disproportionate to its rarity for two reasons. First, the published cases in which localised disease was recognised promptly and completely resected are also those in which the longest survival has been documented, whereas late diagnosis has usually been followed by a poor outcome. This apparent relationship is derived from individual case reports and has not been examined in any comparative or multivariable analysis, and it should therefore be read as a pattern suggested by the literature rather than as an established prognostic effect [8,12,13]. Second, distinguishing a primary seminal vesicle tumor from secondary invasion has direct therapeutic implications, since the management of prostatic, urothelial, and colorectal carcinoma differs fundamentally from that of a seminal vesicle primary [14].

This narrative review consolidates the fragmented literature on primary neoplasia of the seminal vesicle, with emphasis on the diagnostic criteria, the imaging and immunohistochemical features that permit its recognition, the widening spectrum of tumour types, and the current, largely empirical, principles of management. Its principal subject is primary carcinoma of the seminal vesicle, which comprises conventional papillary, mucinous, and clear cell adenocarcinoma together with the rarer squamous cell and neuroendocrine carcinomas. The scope also encompasses those primary seminal vesicle tumours that are neither epithelial nor carcinomatous, namely extragonadal germ-cell tumours, of which seminoma is the reported example [7], mesenchymal malignancies such as sarcoma and the malignant solitary fibrous tumour [8], and the biphasic mixed epithelial and stromal tumour (MEST) [9]. They are retained because they present in exactly the same way as carcinoma, neither imaging nor serum markers separate them from it before tissue is obtained, and they are resolved by the same immunohistochemical panel on core biopsy.

### Literature Search Strategy and Selection Criteria

This article is a narrative rather than a systematic review, and it was not registered or conducted according to PRISMA methodology. A structured but non-systematic literature search was nevertheless performed in PubMed/MEDLINE, Scopus, Web of Science, and Embase, supplemented by Google Scholar for grey literature and by screening of the reference lists of retrieved articles. The search covered the period from 1995 to 2025 and was updated during preparation of the manuscript to include articles published in 2026. Earlier landmark reports, including the original description by Lyons in 1925, were identified from the reference lists of retrieved articles and were retained deliberately, because the total number of reported cases is so small that the earliest reports remain part of the usable evidence base.

The search terms included: “seminal vesicle”; “primary seminal vesicle carcinoma”; “seminal vesicle adenocarcinoma”; and the histology-specific terms “clear cell”, “mucinous”, “papillary”, “squamous”, “neuroendocrine”, “seminoma”, “germ cell tumour”, “sarcoma”, “solitary fibrous tumour”, and “mixed epithelial and stromal tumour”. Further searches combined “seminal vesicle” with “CA-125”, “CK7”, “CK20”, “PAX8”, “NKX3.1”, “immunohistochemistry”, “magnetic resonance imaging”, “core biopsy”, “Zinner syndrome”, “seminal vesicle cyst”, “vesiculectomy”, “radiotherapy”, “chemotherapy”, and “hormonal therapy”, and with “prostate cancer”, “urothelial carcinoma”, “carcinoma in situ”, and “rectal carcinoma” in order to capture the literature on secondary involvement and differential diagnosis.

Publications were eligible if they were written in English, reported histologically confirmed primary neoplasia of the seminal vesicle, or provided data relevant to its diagnosis, differential diagnosis, or management. Case reports, case series, autopsy series, immunohistochemical and molecular studies, imaging series, earlier reviews, and current guideline or classification documents were all included, since no higher level of evidence exists. Where the same case appeared in more than one publication, the most complete report was retained. Selection was performed by the authors by consensus, and priority was given to reports providing histological, immunohistochemical, and outcome data.

The limitations of this evidence base should be stated explicitly, because they constrain every quantitative statement in this review. Approximately one hundred histologically confirmed cases of primary seminal vesicle carcinoma have been reported in the English-language literature in the century since the first description, and the non-epithelial and biphasic neoplasms are represented by no more than a handful of cases each, in some instances by a single report. There are no prospective studies, no randomised comparisons, no population-based incidence data, no disease-specific staging system, and no validated prognostic model. Consequently, the proportions and percentages cited throughout, such as the frequency of papillary architecture, the proportion of tumours expressing CA-125, and the cumulative case counts reported at successive points in time, are descriptive summaries of small, heterogeneous, retrospectively assembled series rather than estimates derived from a defined denominator.

## 2. Anatomy, Embryology, and Histogenesis

Each seminal vesicle is an elongated, lobulated gland approximately 5 cm in length, lying between the bladder and the rectum and draining, via its excretory duct, into the ipsilateral ejaculatory duct after joining the vas deferens. The glandular epithelium is a pseudostratified columnar lining that characteristically contains lipofuscin pigment and displays marked nuclear pleomorphism as a normal, degenerative (“monster cell”) phenomenon—an important pitfall that can be mistaken for malignancy in small biopsies [15].

The mesonephric (Wolffian) origin of the seminal vesicle is central to understanding both its normal biology and the immunophenotype of its tumors. The paramesonephric (Müllerian) ducts, which in the female give rise to the fallopian tubes, uterus, and upper vagina, normally regress in the male but may persist as remnants such as the prostatic utricle or as Müllerian duct cysts. This dual embryological heritage explains two recurring themes in the pathology of seminal vesicle carcinoma: the expression of the Müllerian-associated marker CA-125 by tumors of an ostensibly Wolffian organ [13,16], and the striking, increasingly reported occurrence of clear cell adenocarcinomas showing “ovarian homology,” presumed to arise from Müllerian remnants [17,18]. Congenital anomalies of the mesonephric system—most notably ipsilateral renal agenesis and seminal vesicle cysts, together comprising the Zinner syndrome—have been observed in association with seminal vesicle neoplasia [19,20].

## 3. Epidemiology and Etiology

Because of the rarity of the disease, its true incidence is unknown and cannot be estimated reliably from the available case-based literature. Benson and colleagues assembled twelve acceptable cases spanning 1956 to 1984 [21]; by 2002 Thiel and Effert had collated roughly 52 cases [17]; and by 2021 the cumulative total documented by Campobasso and others had reached approximately sixty [22]. Finally, according to Franco-Buenaventura et al. there are approximately 100 cases reported globally [7]. The reported age at diagnosis is strikingly broad, ranging from 19 to 90 years, with most cases clustering after the age of 50 [17,23]. Notably, several of the histological subtypes—particularly clear cell adenocarcinoma—have been reported in younger men, including patients in their twenties and thirties [2,16,24].

No definite etiological or risk factors have been established. Chronic obstruction and inflammation of the seminal vesicle, long-standing seminal vesicle cysts, and mesonephric maldevelopment have all been proposed as potential predisposing conditions on the basis of individual reports. A case of adenocarcinoma arising in a patient with an 18-year history of a seminal vesicle cyst raised the possibility of malignant transformation within a pre-existing cystic lesion and prompted the recommendation that such cysts be kept under long-term surveillance [18]. Similarly, the recurrent association with renal agenesis and Zinner syndrome suggests that developmental anomalies of the Wolffian and Müllerian systems may create a substrate for later neoplasia [11,19,20]. These associations, however, rest on small numbers and should be regarded as hypothesis-generating rather than established. Practical guidance on surveillance of seminal vesicle cysts and of Zinner syndrome, including when cross-sectional imaging or biopsy is warranted, is consolidated in Section 11.

Elevated serum CA-125 levels are frequently observed in patients with seminal vesicle carcinoma, supporting the concept of a shared embryological origin between the male and female reproductive tracts. The precise histogenesis of seminal vesicle clear cell adenocarcinoma (CCA), however, remains uncertain. Based on its morphological and immunohistochemical resemblance to clear cell adenocarcinomas of the female genital tract, several authors have proposed that this rare neoplasm may arise from persistent Müllerian duct remnants rather than from the seminal vesicle epithelium itself [16,24].

## 4. Clinical Presentation

The clinical hallmark of primary seminal vesicle carcinoma is the absence of specific early symptoms. The gland’s deep pelvic location allows a tumor to grow substantially before producing any complaint, so that the majority of patients present with locally advanced disease [23]. When symptoms do appear, they reflect either local mass effect or invasion of adjacent structures and include hematospermia, hematuria, dysuria and other obstructive lower urinary tract symptoms, perineal or pelvic pain, painful ejaculation, and, occasionally, azoospermia or infertility [25]. Hematospermia in particular, although most often benign in origin, is a recurrent presenting feature and should prompt careful evaluation when it is persistent [7,26].

Digital rectal examination may reveal a palpable, often firm or fixed retrovesical mass and is the single physical sign most likely to indicate a seminal vesicle tumor, detectable in up to roughly one third of patients [10,27]. A minority of tumors are discovered incidentally during imaging performed for unrelated indications, and these fortuitously detected, still-localised lesions represent the subgroup most amenable to curative resection [13].

## 5. Radiological Evaluation

Transrectal ultrasound (TRUS) is a valuable imaging modality for evaluating the seminal vesicles. It provides high resolution visualization, enabling the detection of abnormalities such as asymmetry, enlargement, cystic changes, or solid masses. In cases of seminal carcinoma, TRUS may reveal an irregular hypoechoic or heterogeneous lesion with distortion of the normal seminal vesicle architecture. Although TRUS is useful for lesion detection and for guiding targeted biopsy, magnetic resonance imaging (MRI) remains the preferred modality for local staging and assessing tumor extent due to its superior soft tissue contrast [10,28].

Two adjuncts to conventional greyscale TRUS deserve mention, although neither has been formally evaluated in seminal vesicle neoplasia. Ultrasound elastography, whether strain-based or shear-wave, quantifies tissue stiffness and may help to distinguish a solid neoplastic mass from an inspissated or haemorrhagic cyst, a distinction that greyscale imaging alone does not always resolve [29]. Contrast-enhanced TRUS, using microbubble agents, demonstrates the vascularity of a lesion in real time and can identify enhancing solid components within a predominantly cystic mass, which is of practical value in directing the biopsy needle towards diagnostic rather than necrotic or purely cystic tissue [28,30]. Both techniques are used routinely in prostatic imaging and are readily transferable to the seminal vesicle, but the evidence supporting them at this site is entirely extrapolated and neither should displace MRI for local staging [29,30].

Computed tomography (CT) is usually the first cross-sectional examination to identify and stage a suspected seminal vesicle malignancy. On contrast-enhanced CT, the lesion is typically centered in the retrovesical seminal vesicle region and may appear as a solid, predominantly cystic, or mixed cystic-solid mass. Solid components are commonly heterogeneous and enhance after intravenous contrast administration, whereas areas of hemorrhage, necrosis, mucin, or pre-existing cystic change may produce variable attenuation and internal complexity [13,16,18]. CT is particularly valuable for documenting tumor size, displacement or loss of the normal seminal vesicle contour, involvement of the prostate or bladder base, ureteric obstruction and hydronephrosis, rectal or sigmoid invasion, pelvic or retroperitoneal lymphadenopathy, and distant metastases, especially in the lungs and liver. Multiplanar reformations can help determine whether the bulk of the tumor is centered on the seminal vesicle rather than an adjacent organ and are useful for surgical planning; however, this anatomical inference is not sufficiently specific to establish the primary site [24,31]. Reported cases illustrate a wide spectrum, including enhancing mural nodules within longstanding seminal vesicle cysts, small mildly enhancing cyst-like lesions, and large infiltrative masses inseparable from the prostate, bladder, or bowel. Accordingly, CT findings should be interpreted with MRI, endoscopic assessment where indicated, and histopathological confirmation [32,33]

Magnetic resonance imaging (MRI) is the imaging modality of choice for the evaluation of seminal vesicle carcinoma because of its excellent soft tissue contrast and multiplanar capability. MRI provides detailed assessment of the seminal vesicles, adjacent pelvic structures, and the local extent of disease. Tumors are commonly lobulated and heterogeneous, and may show papillary or cystic-necrotic components with solid enhancing nodules [18,31]. Seminal vesicle carcinoma typically appears as an infiltrative mass with low to intermediate signal intensity on T1-weighted images, heterogeneous intermediate to high signal intensity on T2-weighted images, restricted diffusion on diffusion-weighted imaging (DWI), and heterogeneous enhancement following contrast administration. MRI is also highly valuable for detecting invasion of surrounding organs, neurovascular structures, and regional lymphadenopathy, making it essential for accurate staging, treatment planning, and follow-up [33,34,35].

Positron emission tomography/computed tomography (PET/CT), most commonly performed with ^18F-fluorodeoxyglucose (FDG), has a complementary role in the evaluation of seminal vesicle carcinoma, although its use is limited by the rarity of this malignancy and the absence of standardized imaging guidelines. Primary seminal vesicle carcinomas generally demonstrate increased FDG uptake, reflecting their metabolic activity, which facilitates identification of the primary lesion and assessment of regional lymph node involvement and distant metastatic disease. FDG PET/CT is particularly valuable for whole-body staging, detecting occult metastatic disease that may not be apparent on conventional imaging, and monitoring response to chemotherapy or radiotherapy [13,36]. In the post-treatment setting, PET/CT aid in the early detection of recurrent disease by identifying metabolically active lesions before significant anatomical changes become evident on CT or MRI. Given the limited number of reported cases, the current evidence supporting PET/CT in seminal vesicle carcinoma is derived primarily from case reports and small case series, but the available literature suggests that it provides valuable metabolic information that complements MRI for staging, restaging, and surveillance [36,37].

A structured radiological report should document the form, location, and size of the tumor, the tumor margins, and the presence or absence of prostatic, vesical, or rectal invasion and of suspicious lymphadenopathy. Importantly, imaging cannot reliably distinguish a primary seminal vesicle carcinoma from secondary invasion, nor a malignant lesion from the several benign seminal vesicle entities—cystadenoma, cystic papillary adenoma, and mixed epithelial and stromal tumour—that can mimic it, so tissue diagnosis remains essential [38,39,40].

## 6. Biomarkers and Immunohistochemical Profile

No serum biomarker is specific for primary seminal vesicle carcinoma, making laboratory investigations primarily supportive rather than diagnostic. Serum prostate-specific antigen (PSA) and prostate-specific acid phosphatase (PAP) are typically within normal limits, a finding that is particularly useful because it helps distinguish seminal vesicle carcinoma from prostate cancer [22,23]. Similarly, serum CA19-9 is generally not elevated [23].

Among the available serum markers, CA-125 is the biomarker most consistently associated with seminal vesicle adenocarcinoma. Increased serum concentrations have been documented in a substantial proportion of reported patients and are consistent with the Müllerian-associated immunophenotype observed in many of these tumors, although CA-125 is neither universally elevated nor specific for seminal vesicle origin [15,17,23]. A raised level may therefore support the diagnosis only when interpreted alongside imaging, histopathology, and immunohistochemistry. In the systematic study by Ormsby and colleagues, three of four seminal vesicle adenocarcinomas expressed CA-125, whereas 40 prostatic adenocarcinomas, 32 bladder transitional cell carcinomas, and 10 rectal adenocarcinomas lacked diffuse expression [15]. Serial serum measurements may also provide a useful patient-specific surveillance marker: reported concentrations have fallen after effective treatment and increased with persistent, recurrent, or metastatic disease, in some cases before recurrence became clinically apparent [12,23,40].

Carcinoembryonic antigen (CEA) has a limited role in the evaluation of seminal vesicle tumors. A normal serum CEA concentration cannot be taken as evidence against a colorectal primary or against colorectal invasion of the seminal vesicle, because CEA is raised in only a proportion of colorectal cancers and its concentration varies with stage, tumour differentiation, and smoking status; it therefore has no role in excluding a colorectal origin. Normal seminal vesicle epithelium is generally negative for CEA expression, indicating that CEA is not a physiological marker of this tissue. In contrast, CEA immunoreactivity has been reported in some cases of primary seminal vesicle adenocarcinoma, although its expression is variable and not consistently observed across all tumors [11,41]. Consequently, CEA lacks the sensitivity and specificity required to serve as a reliable diagnostic marker for seminal vesicle malignancies [15]. Instead, it is best regarded as an adjunct immunohistochemical marker that should be interpreted alongside other markers, together with the tumor’s histopathological and clinical features to establish an accurate diagnosis [23,42].

Immunohistochemical markers such as CK7, PAX8, and NKX3.1 play an important role in the diagnosis of seminal vesicle tumors and in distinguishing primary seminal vesicle adenocarcinoma from malignancies originating in adjacent organs. Primary seminal vesicle adenocarcinomas typically express CK7 and PAX8 positivity, supporting their Müllerian-like differentiation, while they are generally negative for NKX3.1, a highly sensitive and specific marker of prostatic origin. Therefore, the immunophenotypic profile of CK7-positive/PAX8-positive/NKX3.1-negative favors a primary seminal vesicle carcinoma over prostate adenocarcinoma invading the seminal vesicles [16,43,44].

Finally, there is a reference to an elevated CA15-3 in a patient with primary SV adenocarcinoma [23]. Recent reports also demonstrate that patients with seminal vesicle tumors may have entirely normal serum tumor markers despite malignant disease, emphasizing that blood biomarkers should always be interpreted alongside imaging and pathological evaluation.

## 7. Core Biopsy

Histopathological confirmation is essential for the diagnosis of primary seminal vesicle carcinoma (PSVC), and transrectal ultrasound (TRUS)-guided core needle biopsy has become the preferred initial tissue sampling technique for patients with a radiologically suspicious seminal vesicle mass [16,45]. Compared with fine-needle aspiration, core biopsy preserves tissue architecture, allowing assessment of papillary or glandular morphology while providing sufficient material for an extensive immunohistochemical (IHC) panel. This is particularly important because the principal diagnostic challenge is not identifying malignancy, but determining whether the tumor originates from the seminal vesicle or represents secondary invasion from the prostate, bladder, or colorectum. In the case reported by Bhardwaj et al., the diagnosis of primary seminal vesicle adenocarcinoma was established directly from a TRUS-guided core biopsy using this immunohistochemical panel and was subsequently confirmed in the surgical specimen, demonstrating that accurate diagnosis is achievable before definitive surgery [45].

TRUS guidance is not the only option, and it is not always the best one. Lesions that lie superiorly or laterally within the seminal vesicle, that are small, or that are obscured by a large cystic component may be poorly visualised transrectally, and in these circumstances MRI-guided in-bore biopsy, or software fusion of the diagnostic MRI with real-time ultrasound, allows the needle to be directed at the enhancing solid component identified on the staging study [28,45]. Fusion targeting is now routine in prostatic practice and requires no additional hardware beyond that already present in most units; its application to the seminal vesicle has not been studied systematically but follows the same principle, namely that the tissue sampled should be the tissue that generated the radiological abnormality. The procedure is not without risk, and this should be discussed with the patient. Transrectal needle biopsy carries the recognised complications of haematuria, haematospermia, rectal bleeding, and infection ranging from transient fever to sepsis, the last being the principal reason for the growing preference for a transperineal route in prostatic practice [46]. These considerations reinforce the general principle that biopsy should be undertaken when its result will alter management, and that the approach should be selected to give the shortest and safest path to the target.

Despite its diagnostic value, core biopsy has important limitations. Primary seminal vesicle tumors may be heterogeneous, and limited samples may not capture the characteristic architecture, an in-situ component, or the most diagnostically informative areas. Poorly differentiated carcinomas may also lose lineage-associated markers, while small samples from biphasic or mesenchymal lesions may establish the broad tumor category without reliably determining malignant potential [9,15]. Core-biopsy findings should therefore be correlated with the anatomical center and extent of the lesion on CT and MRI and interpreted using an appropriately broad immunohistochemical panel. When imaging and biopsy findings are discordant or classification remains uncertain, complete excision and comprehensive examination of the resection specimen remain the definitive diagnostic approach [18,44,47].

## 8. Diagnostic Criteria and the Problem of Exclusion

Primary seminal vesicle carcinoma (PSVC) is an exceptionally rare malignancy, and its diagnosis remains one of exclusion because secondary invasion of the seminal vesicles by prostate, bladder, or colorectal carcinomas is far more common. The exclusion required is twofold: a secondary carcinoma of adjacent origin must be ruled out, and the non-epithelial and biphasic primary neoplasms must be excluded before a lesion is classified as a carcinoma of seminal vesicle origin. Although the original criteria proposed by Dalgaard and Giertsen relied mainly on tumor location and morphology, current diagnosis integrates clinical assessment, imaging, histopathology, and immunohistochemistry [2]. No single morphological or immunohistochemical marker is diagnostic; therefore, a combination of findings is required to establish the diagnosis (Table 1).

Immunohistochemistry is the linchpin of the diagnosis of primary seminal vesicle carcinoma, both for confirming a seminal vesicle origin and for excluding the commoner tumours that secondarily involve the gland. The characteristic immunophenotype of papillary seminal vesicle adenocarcinoma is positivity for CA-125 and CK7, negativity for CK20, and negativity for the prostatic markers PSA and PAP [48]. The coordinate CK7 and CK20 profile provides a useful orientation but should not be treated as decisive. CK20 is expressed in only about half of urothelial carcinomas, so that a CK7-positive and CK20-negative result does not exclude urothelial carcinoma; and although a CK7-negative and CK20-positive profile is typical of colorectal adenocarcinoma, exceptions occur in both directions. Negative PSA and PAP argue against prostatic adenocarcinoma but do not settle the question in poorly differentiated tumours [49,50]. Additional markers reported in seminal vesicle adenocarcinoma include PAX8 and MUC6. PAX8 expression is, however, variable rather than defining: it is a developmental transcription factor of the Wolffian and Müllerian systems that is also expressed in thyroid, renal, and gynaecological tumours, and polyclonal antibodies cross-react with other members of the PAX family, so a positive result is supportive only in the appropriate anatomical and morphological context while a negative result does not exclude a seminal vesicle origin [51,52]. CEA is variable and more often negative than positive.

Urothelial carcinoma warrants particular emphasis among the tumours that must be excluded, because bladder cancer is the tenth most frequently diagnosed malignancy worldwide and is therefore, in absolute terms, the commonest source of a carcinoma involving the seminal vesicle [53]. In bladder cancer, cystoscopy remains essential for evaluating a urothelial origin; therefore, a positive urine cytology should prompt formal cystoscopic assessment with directed and, when indicated, random biopsies.

For the tissue diagnosis itself the coordinate CK7 and CK20 profile is a starting point rather than a solution, and a lineage-specific panel is required. Urothelial differentiation is supported by GATA3, p63, uroplakin II, and S100P. GATA3 is the most useful single discriminator, although its sensitivity should not be overstated: a meta-analysis of urothelial carcinoma reported a pooled expression rate of approximately 75%, falling to about 61% in upper tract tumours, so that a negative GATA3 result does not exclude urothelial carcinoma [54]. It is nevertheless essentially absent from prostatic adenocarcinoma, and a GATA3-positive, NKX3.1-negative profile indicates urothelial rather than prostatic origin [55,56]. Its limitations should nevertheless be recognised: GATA3 is not organ-specific, being expressed in breast, salivary gland, and mesonephric lesions among others, and expression may be diminished in poorly differentiated and variant urothelial carcinomas [57]. S100P and the basal markers p63 and p40 are useful complements; p40 is the more specific of the two, and the combination of S100P with p63 labels the great majority of urothelial carcinomas [55]. Uroplakin II is considerably more specific for urothelium than GATA3 but less sensitive, so that a positive result is close to confirmatory while a negative result is uninformative [58]. Within the urothelium, aberrant full-thickness CK20 expression, abnormal p53 staining in the form of strong diffuse positivity or complete loss, and reduced or absent CD44 identify carcinoma in situ and distinguish it from reactive atypia; awareness of pagetoid spread, in which isolated malignant cells lie within otherwise unremarkable urothelium, prevents a limited sample from under-representing urothelial disease [53].

Where a colorectal primary is in question, CDX2 and SATB2 should be used rather than CK20 alone: SATB2 is more specific than CK20, retains its expression at metastatic sites, and in combination with CDX2 gives the best discrimination of colorectal origin [59]. Seminal vesicle adenocarcinoma, by contrast, is negative for GATA3, p63/p40, uroplakin II, CDX2, and SATB2, and it is the combination of CA-125 and/or PAX8 expression with an absence of urothelial, prostatic, and intestinal lineage markers that supports a seminal vesicle origin. No individual marker is decisive in either direction; the panel is interpreted as a whole, against the morphology and the anatomical centre of the lesion, and false-positive and false-negative results occur with every constituent [15,16,53].

Two important caveats temper this scheme. First, in poorly differentiated tumors CA-125 may be lost—the single poorly differentiated seminal vesicle adenocarcinoma in the Ormsby series was CA-125-negative—so a negative CA-125 result does not exclude the diagnosis [12,15]. Second, a very small proportion of poorly differentiated prostatic adenocarcinomas lose both PSA and PAP, occasionally blurring the distinction from a seminal vesicle primary; in such cases tumor location, histomorphology, and serum PSA must be integrated with the stains [15]. The comparative immunohistochemical profiles that underpin the differential diagnosis are set out in Table 2.

## 9. Pathology and the Spectrum of Primary Seminal Vesicle Neoplasms

Adenocarcinoma remains the dominant epithelial malignancy, but the principal biological message is the diversity of differentiation that can occur in the seminal vesicle. Conventional and papillary tumours broadly retain the gland’s characteristic epithelial phenotype, whereas mucinous and clear-cell lesions point to biologically distinct pathways. In particular, Müllerian-type differentiation and the occurrence of non-glandular epithelial, germ-cell, mesenchymal, and biphasic tumours challenge any definition based on papillary morphology alone. Detailed pathological, immunohistochemical, and clinical features of each entity are summarised in Table 3; the narrative below therefore emphasises the biological and diagnostic implications rather than repeating those tabulated features.

### 9.1. Papillary and Conventional Adenocarcinoma

Papillary architecture represents the classical morphological expression of seminal vesicle adenocarcinoma and underlies the historical Dalgaard–Giertsen criteria and much of the established CA-125/CK7 immunophenotypic literature [15]. Its main biological significance is that loss of differentiation can be accompanied by loss of lineage-associated markers, particularly CA-125, making poorly differentiated tumours less securely classified by phenotype alone [12,15]. Detailed morphology and diagnostic features are summarised in Table 3.

### 9.2. Mucinous Adenocarcinoma

Mucinous adenocarcinoma is important less for its individual microscopic details, which are summarised in Table 3, than for the evidence that mucinous lesions may constitute a biologically distinct subgroup. Their often cystic appearance also links tumour biology to a practical diagnostic pitfall: a seminal-vesicle cystic lesion cannot be assumed to be benign solely from imaging or age at presentation [13]. This distinction has acquired additional relevance with the identification of activating KRAS mutations in a subset of mucinous cystic tumours, discussed in Section 10.5 [60].

### 9.3. Clear Cell Adenocarcinoma and Müllerian/Ovarian Homology

Clear cell adenocarcinoma provides the strongest morphological evidence of Müllerian-type differentiation in this region. Its resemblance to female-genital-tract clear cell carcinoma, frequent PAX8 expression, and reported ‘ovarian homology’ support the concept that persistent Müllerian remnants may contribute to seminal-vesicle tumorigenesis [16,24]. Biologically, these tumours demonstrate that primary seminal-vesicle carcinoma is not a single uniform Wolffian epithelial entity and that historical papillary criteria are insufficient for the full contemporary spectrum. The detailed pathological and clinical characteristics are provided in Table 3.

### 9.4. Squamous Cell and Neuroendocrine Carcinoma

Squamous cell and poorly differentiated neuroendocrine carcinomas broaden the epithelial spectrum beyond gland-forming malignancy [61,62]. Their significance is conceptual as well as diagnostic: both are true carcinomas of the seminal-vesicle region, yet neither is expected to reproduce the classical papillary morphology or conventional CA-125-positive phenotype. Their existence reinforces the need to establish lineage and primary site by integrated morphology, immunohistochemistry, and exclusion of another primary rather than by a single historical pattern (Table 3).

### 9.5. Non-Epithelial and Biphasic Primary Neoplasms of the Seminal Vesicle

Non-epithelial and biphasic neoplasms are included because they enter the same pre-biopsy differential diagnosis as carcinoma, although they are biologically distinct. Seminoma represents germ-cell differentiation; sarcoma and malignant solitary fibrous tumour represent mesenchymal lineages; and mixed epithelial and stromal tumour (MEST) is a biphasic epithelial-stromal neoplasm [7,8,9,17]. Their practical importance is that imaging and serum markers do not reliably establish lineage, whereas tissue classification directly changes treatment expectations, particularly for the chemo- and radiosensitive germ-cell tumours. Table 3 contains the entity-specific pathological and clinical details; the overarching point is that a seminal-vesicle mass should be approached as a lineage-classification problem before management is assigned.

**Table 3:** Spectrum of primary seminal vesicle neoplasms. The upper rows list the epithelial subtypes that together constitute primary seminal vesicle carcinoma. The final three rows (seminoma, sarcoma, and mixed epithelial and stromal tumour) are non-epithelial or biphasic neoplasms that are not carcinomas; they are included because they enter the differential diagnosis of a primary seminal vesicle mass and cannot be distinguished from carcinoma on clinical or radiological grounds.

Tumour Type	Key Pathological Features	Immunohistochemical/Diagnostic Features	Clinical Features and Diagnostic Considerations	References
Conventional adenocarcinoma	Most common histological type. Typically demonstrates papillary glandular growth resembling the normal seminal vesicle, prostatic duct adenocarcinoma of Gleason pattern 3–4, or mucinous carcinoma. Higher-grade tumours may contain poorly differentiated areas.	An in-situ component in adjacent non-neoplastic seminal vesicle epithelium strongly supports a primary seminal vesicle origin.	Must be distinguished from carcinomas arising in adjacent organs, particularly the prostate, bladder, rectum, and kidney.	[15,23,51]
Papillary adenocarcinoma	Prototypical form of primary seminal vesicle carcinoma. Usually well differentiated, with prominent papillary architecture. Approximately 90% of reported seminal vesicle adenocarcinomas demonstrate this pattern.	Commonly CA-125 positive. The classical Dalgaard–Giertsen morphological criteria and the immunophenotypic findings described by Ormsby and colleagues are based largely on this subtype.	The standard diagnostic immunohistochemical panel performs best in well-differentiated tumors. Poorly differentiated variants may lose CA-125 expression and become difficult to distinguish from morphological mimics.	[12,15]
Mucinous (colloid) adenocarcinoma	Characterised by abundant extracellular mucin and may appear predominantly cystic.	Serum CA-125 may be elevated, while PSA, PAP, and CEA can remain within normal limits.	May closely mimic a benign seminal vesicle cyst on imaging.	[13]
Clear cell adenocarcinoma	Histologically resembles clear cell carcinoma of the female genital tract, particularly ovarian clear cell carcinoma. It may not demonstrate the classical papillary architecture associated with conventional seminal vesicle adenocarcinoma.	Frequently expresses Müllerian and clear-cell-associated markers, including PAX8. Its morphology and immunophenotype may demonstrate “ovarian homology.”	Thought to arise from Müllerian remnants within or adjacent to the seminal vesicle. Reported in relatively young or middle-aged men, often presenting with haematospermia and haematuria. It may recur early and can be misdiagnosed as metastatic renal cell carcinoma.	[16,24,32]
Squamous cell carcinoma	Primary malignant squamous differentiation involving the seminal vesicle.	Diagnosis requires exclusion of direct extension or metastasis from another primary site.	Exceptionally rare	[61]
Poorly differentiated neuroendocrine carcinoma	High-grade, poorly differentiated neuroendocrine morphology.	Neuroendocrine marker expression is expected	Extremely rare, aggressive, and associated with poor prognosis.	[62]
Primary seminal vesicle seminoma	Germ-cell tumour with seminomatous morphology arising in the seminal vesicle.	Requires confirmation of germ-cell differentiation and exclusion of a gonadal primary tumour.	Represents an exceptionally rare extragonadal germ-cell tumour	[7]
Sarcomas	Mesenchymal malignant tumours arising from the stromal tissues of the seminal vesicle.	Diagnosis depends on the specific line of mesenchymal differentiation.	Rare and cannot be reliably predicted from clinical presentation or imaging findings alone.	[9,17]
Mixed epithelial and stromal tumour (MEST)	Low-grade biphasic neoplasm composed of epithelial and mesenchymal or stromal elements. It may form a large mass.	Recognition of the characteristic biphasic epithelial–stromal architecture is central to diagnosis.	Despite potentially large size, it may follow an indolent clinical course. Its appearance reinforces the need for histological examination rather than reliance on imaging alone.	[9,17]

## 10. Management

Because primary seminal vesicle carcinoma is exceptionally rare, no prospective trials, validated staging framework, or evidence-based treatment guideline exists. Management is consequently derived from isolated case reports, small retrospective series, and historical reviews, and must be individualised according to tumour extent, histological subtype, resectability, previous treatment, and patient fitness. Discussion within a multidisciplinary genitourinary oncology team is particularly important because treatment may require complex pelvic surgery, radiotherapy, systemic therapy, and histology-specific input [17,23,63].

### 10.1. Surgical Management

Complete surgical excision is the treatment most frequently reported in patients who achieved durable control of localised disease, and it is accordingly regarded as the cornerstone of management, although no comparative data exist to quantify its benefit relative to other approaches. The operative objective is an en-bloc complete resection with microscopically negative margins (R0 resection), because incomplete excision and residual pelvic disease are repeatedly associated with recurrence and the need for additional treatment [10,11,12]. The extent of surgery should be adapted to the anatomical distribution of the tumor rather than determined by a uniform procedure [21,23,64].

For small, apparently organ-confined tumors, isolated seminal vesiculectomy may be adequate, provided that negative margins can be achieved. Tumors involving or densely adherent to the prostate generally require radical prostato-vesiculectomy, while invasion of the bladder, distal ureter, rectum, or sigmoid colon may necessitate cystoprostatectomy, ureteric reconstruction, bowel resection, or pelvic exenterative surgery. Pelvic lymph-node dissection is frequently included for staging or when radiologically suspicious nodes are present, but neither its optimal template nor a prophylactic therapeutic benefit has been established [7,17,23,32].

Surgery is technically demanding because the seminal vesicles occupy a deep and confined retrovesical space adjacent to the prostate, bladder, ureters, rectum, neurovascular bundles, and pelvic floor. Open surgery remains appropriate for bulky or multivisceral disease, whereas laparoscopic and robot-assisted techniques have been successfully used for selected localised tumors. The magnified view and instrument articulation of robotic surgery may facilitate precise dissection in this narrow compartment, but the published evidence consists of individual cases and does not establish oncological superiority over open or conventional laparoscopic approaches [16,18,24,38]. The choice of approach should therefore be determined by the characteristics of the tumour and by the experience of the operating surgeon, and not by a general preference for one technique over another. Tumour size, the presence and extent of invasion of adjacent organs, the need for reconstruction, and previous pelvic surgery or radiotherapy all bear on the decision, as does the volume of comparable pelvic work performed by the surgeon and the unit. A minimally invasive approach is appropriate only where an R0 resection can be achieved with the same confidence as at open surgery; where it cannot, conversion or a planned open procedure is the correct decision rather than a failure of technique.

### 10.2. Adjuvant Radiotherapy

The role of postoperative radiotherapy remains empirical because comparative studies are lacking. It has most often been used after incomplete resection, for positive or close margins, extracapsular or adjacent-organ extension, residual pelvic tumor, and occasionally for local recurrence [10,11,12]. Reported postoperative regimens include approximately 60 Gy in 30 fractions and 66 Gy in 30 fractions. Durable control has been described after combined surgery and radiotherapy, but surgery-alone survivors have also been reported after complete resection; routine irradiation after an R0 excision therefore cannot be recommended from the available evidence [17,18,23].

### 10.3. Systemic Chemotherapy

Chemotherapy is generally reserved for unresectable, recurrent, or metastatic disease, but it also has a defensible role before surgery in selected patients with locally advanced tumours. No regimen is standard. Published treatments include paclitaxel-cisplatin, gemcitabine-cisplatin, cisplatin-etoposide, docetaxel-based therapy, FOLFOX or other fluoropyrimidine-oxaliplatin combinations, and dose-dense methotrexate-vinblastine-doxorubicin-cisplatin (MVAC). Responses range from rapid progression to prolonged remission, and regimen selection has usually been extrapolated from urothelial, colorectal, ovarian-type, neuroendocrine, or germ-cell malignancies according to morphology and clinical context [7,12,23,63,65,66].

The strongest single argument for a neoadjuvant strategy is the most striking outcome reported in this disease. A patient with pulmonary metastatic adenocarcinoma received dose-dense MVAC, which produced sufficient response to permit radical pelvic surgery and subsequent pulmonary metastasectomy, and complete remission was maintained for 48 months [23]. The relevance of this report extends beyond the individual outcome. It demonstrates that a seminal vesicle adenocarcinoma may be chemosensitive, that response can convert disease that was not initially operable into disease that can be resected with negative margins, and that this sequence is compatible with prolonged remission even in the presence of distant metastasis at presentation. Since complete excision is the treatment most consistently associated with durable control, and since a substantial proportion of these tumours are locally advanced when first identified, a neoadjuvant regimen offered with the explicit intention of achieving resectability is a rational strategy in a patient who is fit for both the chemotherapy and the subsequent operation. The evidence remains a single case, the choice of regimen must still be guided by histological subtype and by the response patterns of morphologically analogous tumours, and no response rate can be quoted; the strategy should therefore be discussed within a multidisciplinary team and the patient informed of the basis on which it is offered. Other reports describe temporary or partial responses to platinum-based or multiagent chemotherapy, sometimes combined with androgen deprivation, but disseminated disease generally remains difficult to control [63,65,66]. These observations support consideration of aggressive multimodal treatment in carefully selected patients while underscoring that the evidence is anecdotal [61].

### 10.4. Hormonal Therapy

Hormonal therapy has been used on the assumption that some seminal vesicle adenocarcinomas may retain androgen responsiveness. Historical treatments include oestrogens, orchiectomy, anti-androgens, and luteinising hormone-releasing hormone agonists, either as adjuvant treatment or palliation for advanced disease. Individual cases have shown biochemical or clinical stabilisation, including responses when hormonal treatment was combined with chemotherapy, but the predictive value of androgen-receptor expression, the optimal agent, and treatment duration remain undefined. Endocrine therapy should therefore be considered an individualised option rather than a standard component of care (Table 4) [17,63,65].

### 10.5. Emerging Considerations

Molecular characterisation of seminal vesicle neoplasia is at an early stage, but it is no longer absent, and the alterations so far identified are of a kind that carries therapeutic implications. The most substantial contribution is the genomic analysis of primary epithelial neoplasms of the seminal vesicle by Collins and colleagues, which identified a distinct subset of mucinous cystic tumours driven by activating KRAS mutations [60]. The finding matters for two reasons. It supports the separation of mucinous lesions from conventional adenocarcinoma as a biologically distinct group rather than a purely morphological variant, and it places these tumours within the KRAS-mutant category for which direct inhibitors, initially against the G12C allele and subsequently against G12D and pan-RAS targets, are now in clinical use or advanced trials elsewhere. Whether any of these agents has activity at this site is unknown, and no case has been reported; the point is that the alteration is actionable in principle and will be missed unless it is sought.

Mismatch repair and microsatellite instability status has not been systematically assessed in primary seminal vesicle carcinoma, and no estimate of the prevalence of deficient mismatch repair at this site can be given. Testing is nevertheless justified in advanced disease, for two reasons that do not depend on site-specific data. Pembrolizumab is approved on a tumour-agnostic basis for previously treated, unresectable or metastatic solid tumours that are microsatellite-instability-high or mismatch-repair-deficient, an indication derived from the KEYNOTE-158 study, in which an objective response was observed in approximately a third of patients with non-colorectal tumours of this type [67]. A deficient result would therefore open a treatment option in a disease that otherwise has none of established value once surgery is no longer possible. Immunohistochemistry for the four mismatch repair proteins is inexpensive, is available in any diagnostic laboratory, and can be performed on the same block used for the diagnostic panel described in Section 8, so the threshold for requesting it should be low. A deficient result additionally raises the question of Lynch syndrome and of germline testing, with consequences for the patient’s relatives that extend beyond the management of the tumour itself.

Tumour mutational burden is the second agnostic biomarker of practical relevance. Pembrolizumab carries a tumour-agnostic indication for previously treated unresectable or metastatic solid tumours with a burden of at least ten mutations per megabase, based on the prospective biomarker analysis of KEYNOTE-158, in which response was more frequent in the high-burden group [68]. No measurement of tumour mutational burden in a primary seminal vesicle carcinoma has been published. The threshold is assay-dependent and the biomarker is imperfect, with well-recognised debate about the validity of a single universal cut-off across tumour types, but the practical implication for a patient with exhausted conventional options is straightforward: comprehensive genomic profiling that reports mutational burden alongside targetable alterations may identify an approved therapy where none would otherwise exist.

PD-L1 expression is more difficult to interpret and should be approached with greater caution. No data exist for seminal vesicle tumours, and PD-L1 is not a site-independent biomarker: the antibody clone, the scoring system, whether tumour cells alone or the combined positive score is used, and the threshold that defines positivity all differ between tumour types and between the trials that validated them. A PD-L1 result obtained on a seminal vesicle carcinoma therefore cannot be interpreted against the cut-off established for urothelial or any other carcinoma, and it should not by itself be used to justify or withhold a checkpoint inhibitor. Its value at present is descriptive, and reporting it in published cases would help to establish whether any consistent pattern exists.

A practical position follows from these observations. In a patient with unresectable, recurrent, or metastatic disease, we would regard mismatch repair immunohistochemistry as a reasonable minimum, with comprehensive genomic profiling including mutational burden, microsatellite status, and a broad targeted panel where the tissue and the resources permit. Equally important is that the results be published, whether positive or negative, since the absence of negative results is one reason the molecular landscape of this disease remains as poorly defined as it is. Immunotherapy has not been systematically evaluated at this site and the available experience is anecdotal [69]; collaborative registries incorporating central molecular characterisation remain the only realistic route to changing this.

## 11. Prognosis and Follow-Up

The prognosis of primary seminal vesicle carcinoma has historically been poor. Because most tumours present with peripheral infiltration or distant metastasis, the classical teaching is that the disease is usually unresectable and that most patients die within two to three years of diagnosis [17]. Metastatic spread occurs to pelvic, para-aortic, and other lymph nodes and to the lungs and liver, with bone and other sites less commonly involved [23]. Reported times to progression have varied widely, from as little as two months to around twenty-one months [23].

This overall picture appears to differ according to stage at diagnosis, although the relationship can only be inferred from the pattern of published cases. Patients whose tumours were detected while still localised and were completely resected have been reported to achieve prolonged disease-free survival, including a five-year recurrence-free follow-up after surgery, chemotherapy, and radiotherapy [12], and a durable remission at four years in a patient with metastatic disease treated with neoadjuvant chemotherapy, surgery, and metastasectomy [23]. Taken together, these reports are consistent with a relationship between recognition of a still-resectable tumour and longer survival, and this remains the most plausible working assumption for clinical practice. It cannot, however, be presented as an established determinant of prognosis. The available data are uncontrolled and retrospective, staging is not standardised in the absence of a disease-specific system, follow-up is short and inconsistently reported, and individual case reports are subject to selection and publication bias favouring favourable outcomes. No comparative or multivariable analysis of prognostic factors in primary seminal vesicle carcinoma has been performed, and no validated prognostic model exists. The association between early recognition and survival is therefore best described as suggested by the published literature rather than demonstrated by it. Given the propensity for late local recurrence and metastasis, prolonged follow-up with cross-sectional imaging—and, where the tumor expressed it, serial serum CA-125—is prudent [18,40,70].

No follow-up schedule for this disease has been validated, and none can be, for the reasons set out above. What follows is therefore offered as a pragmatic scheme, derived from the observed behaviour of the reported cases and from the schedules used for pelvic malignancies of comparable recurrence risk, and it should be adapted to the individual patient rather than applied uniformly.

Clinical assessment, including digital rectal examination, is reasonable every three to four months for the first two years, every six months to five years, and annually thereafter. Cross-sectional imaging of the abdomen and pelvis should be obtained at the same intervals for the first two years and six-monthly to five years, with MRI preferred for the pelvis because of its superior soft-tissue contrast in the operated retrovesical space and CT preferred for the abdomen and for the assessment of nodal and visceral disease; the chest should be included, since the lung is among the commoner sites of distant relapse. A baseline study three months after surgery is particularly valuable, since the post-operative appearances at that point become the comparator against which all later studies are read.

Where the tumour expressed CA-125 and the serum concentration fell after resection, serial measurement at each clinical visit is worthwhile, since a rise may precede radiological recurrence. Two cautions apply: a normal CA-125 does not exclude recurrence, particularly in poorly differentiated tumours that may have lost expression, and a rise in isolation should prompt imaging rather than treatment. Where the tumour did not express CA-125, the marker has no role in surveillance and should not be requested. FDG-PET/CT is not indicated routinely, but is reasonable where recurrence is suspected clinically or biochemically and conventional imaging is equivocal.

Follow-up should not be discontinued at five years. Late recurrence has been reported, the natural history is poorly characterised, and annual clinical review with imaging determined by symptoms is a proportionate long-term arrangement in a patient who remains fit. Attention should also be given to the sequelae of treatment rather than to oncological surveillance alone, including urinary and sexual function after radical pelvic surgery, bowel and bladder toxicity after radiotherapy, and, where androgen deprivation was used, bone density and metabolic consequences. Finally, long-standing seminal vesicle cysts, and any cyst in a man with Zinner syndrome, warrant continued surveillance in view of the possibility, however rare, of malignant transformation. Population screening of men with Zinner syndrome is not justified on present evidence, but a change in cyst size, the appearance of a solid or enhancing component, new haematospermia or pelvic pain, or a rising serum CA-125 in a patient in whom it was previously normal should prompt cross-sectional imaging and, if a solid component is confirmed, biopsy.

### The Absence of a Staging System and a Proposed Minimum Reporting Dataset

A specific and remediable obstacle, the absence of a staging system for tumours of the seminal vesicle underlies much of the uncertainty described above. Neither the AJCC Cancer Staging Manual nor the UICC TNM classification contains a chapter for this site; the seminal vesicle enters TNM only as a structure invaded by tumours arising elsewhere, most familiarly as category pT3b of prostatic carcinoma. The WHO classification defines the histological entities that arise here but assigns no stage groupings to them. Cancer registries can record the site through the ICD-O topography code C63.7, but extent of disease is captured only as the three-tier localised, regional, or distant summary, which is too coarse to support any analysis of prognosis.

The consequences are apparent throughout the literature. Reports describe extent narratively, in terms that differ from one paper to the next, so that the cases described as organ-confined in one series are not demonstrably comparable with those so described in another. Some authors have applied the T categories of prostatic or bladder carcinoma by analogy, but this practice is unvalidated and is arguably misleading, since the seminal vesicle has no capsule equivalent to that of the prostate and the boundary of organ-confined disease is correspondingly ill-defined. No proposal for a seminal-vesicle-specific TNM classification has been published, and the number of cases available is in any event too small for the conventional route to a staging system, namely derivation and validation in independent cohorts.

A staging system cannot be derived from data that were never collected in comparable form, and the useful first step is therefore not a classification but a reporting standard. We propose the minimum dataset set out in Table 5, which asks only for information already available at the time of surgery and pathological examination and requires no additional investigation. If such a dataset were adopted by those reporting individual cases, and ideally aggregated through an international registry with central pathological review, the accumulated reports of the next decade would become comparable with one another, and the questions that cannot presently be answered, above all whether extent of disease at diagnosis independently predicts survival, would become answerable. Until then, the descriptive statements made throughout this review are the most that the evidence will support.

## 12. Conclusions and Future Directions

**Figure 1:**
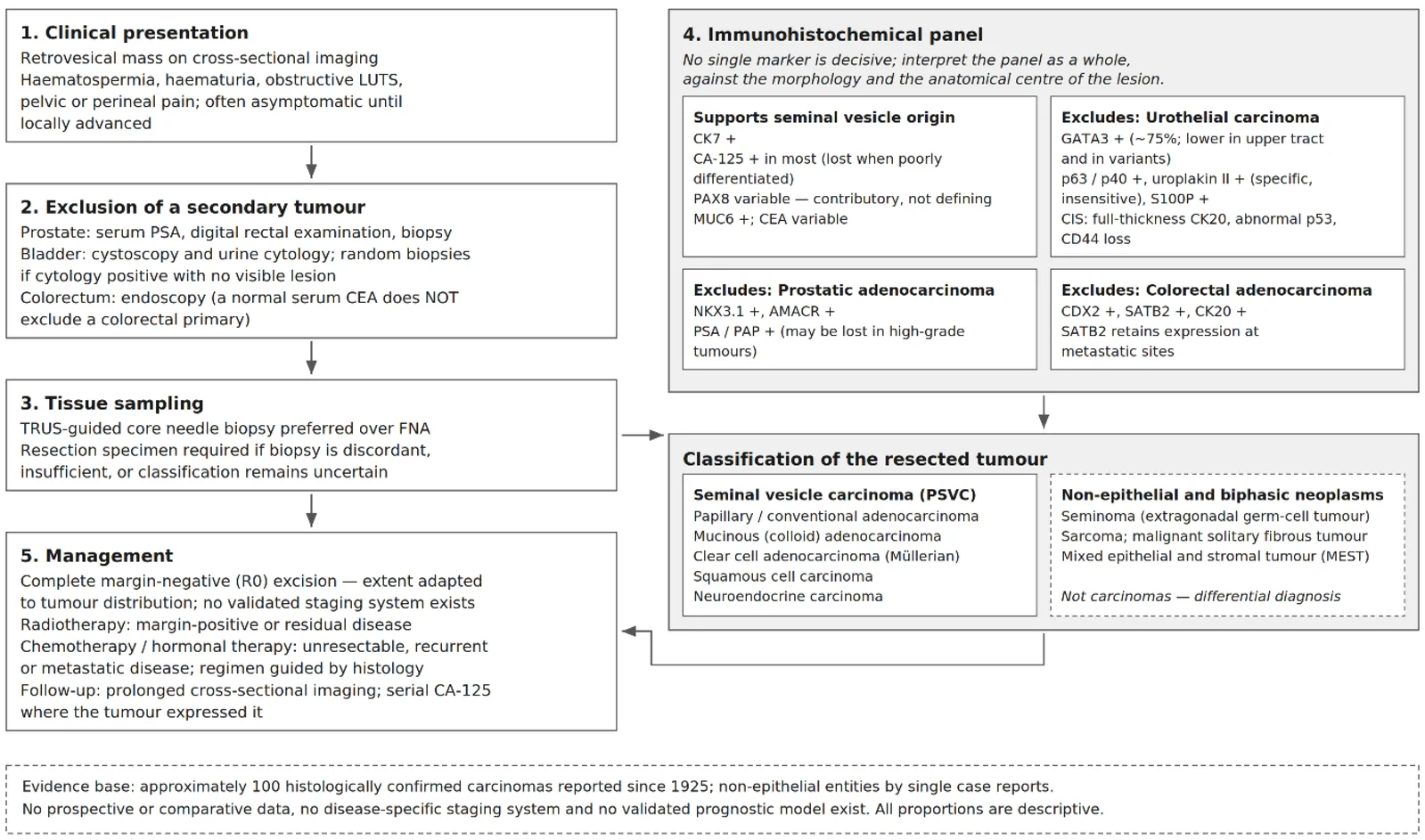
Diagnostic and management algorithm for a suspected primary neoplasm of the seminal vesicle. The left column shows the sequence from clinical presentation through exclusion of a secondary tumour to tissue sampling and management; the right column shows the immunohistochemical panel used to establish lineage and the resulting classification into primary seminal vesicle carcinoma and the non-epithelial and biphasic neoplasms that are not carcinomas (broken outline). Marker entries indicate the usual pattern rather than an invariable one and correspond to the values given in Table 1 and Table 2. Abbreviations: LUTS, lower urinary tract symptoms; TRUS, transrectal ultrasound; FNA, fine-needle aspiration; CIS, carcinoma in situ; R0, microscopically margin-negative resection; PSVC, primary seminal vesicle carcinoma; MEST, mixed epithelial and stromal tumour. Figure prepared by the authors; all elements in the figure are original, and no previously published material is reproduced.

Primary seminal vesicle carcinoma remains a diagnosis of exclusion that requires close integration of clinical assessment, CT and MRI, tissue morphology, and immunohistochemistry. No single serum or tissue marker is diagnostic. In the appropriate anatomical and morphological context, a CK7-positive tumour with CA-125 expression, absence of PSA, PAP, and NKX3.1, and absence of the urothelial and intestinal lineage markers GATA3, p63/p40, uroplakin II, CDX2, and SATB2 supports seminal vesicle origin, while PAX8 expression is contributory rather than defining and CK20 negativity alone does not exclude urothelial carcinoma. Marker loss in poorly differentiated tumours demands cautious interpretation, as does the recognition that not every primary neoplasm of the seminal vesicle is a carcinoma. Alongside the Müllerian-type and neuroendocrine carcinomas, germ-cell, mesenchymal, and biphasic tumours arise at this site and share the clinical and radiological presentation of carcinoma while differing in classification and, importantly, in treatment. Image-guided core biopsy can establish the diagnosis preoperatively, yet discordant or insufficient samples require definitive assessment of the resection specimen (Figure 1).

Complete margin-negative excision is the treatment most consistently associated with durable control of localised disease in the reported cases and is accordingly regarded as the principal potentially curative option. Radiotherapy is most defensible for residual or margin-positive tumour, while chemotherapy and hormonal therapy are reserved largely for unresectable, recurrent, or metastatic disease and should be selected according to histology and individual clinical circumstances. The apparent improvement in outcomes among recently reported, completely resected cases is consistent with a benefit from early recognition and accurate localisation, but the anecdotal and uncontrolled nature of this evidence means that the association is suggested rather than established, and no prognostic factor in this disease has yet been validated. Progress will depend on international case registries, central pathological review, standardised reporting of stage and treatment, and genomic profiling capable of identifying biologically distinct subgroups and actionable alterations. A necessary precondition is the absence, at present, of any staging system for this site in either the AJCC or the UICC classification; the minimum reporting dataset proposed in Table 5 is offered as a practicable first step, since comparable data must be accumulated before a staging system or a prognostic model can be derived at all. Until such evidence is available, prolonged surveillance and multidisciplinary, individualised management remain essential.

## Figures and Tables

**Table 1:** Current diagnostic criteria for primary seminal vesicle carcinoma (PSVC). PSA, prostate-specific antigen; PAP, prostatic acid phosphatase; CK, cytokeratin.

Diagnostic Criterion	Typical Findings in Primary Seminal Vesicle Carcinoma (PSVC)	Diagnostic Value
Clinical presentation	Hematospermia, hematuria, dysuria, pelvic/perineal pain, painful ejaculation, obstructive lower urinary tract symptoms	Nonspecific; raises clinical suspicion
Radiological findings	Mass centered in the seminal vesicle on MRI/CT, with possible invasion of adjacent structures	Defines tumor origin, local extent, and resectability
Exclusion of another primary tumor	No evidence of primary prostate, bladder, colorectal, or other pelvic malignancy	Essential diagnostic criterion
Histopathology	Adenocarcinoma, commonly papillary or tubulopapillary architecture; may demonstrate in situ component	Supports diagnosis but is not specific
Serum biomarkers	PSA and PAP usually normal; CA-125 may be elevated; CEA and CA19-9 are variable and nonspecific	Supportive but not diagnostic
Immunohistochemistry	CK7+, CA-125+ in most; PAX8 variable; PSA−, PAP−, NKX3.1−; CK20 usually negative. A urothelial and intestinal panel (GATA3, p63/p40, uroplakin II, CDX2, SATB2) is required, since CK20 negativity alone does not exclude urothelial carcinoma	Most useful tool for determining seminal vesicle origin, but supportive rather than decisive; no single marker is diagnostic
Clinicopathological correlation	Integration of clinical findings, imaging, histopathology, immunophenotype, and exclusion of other primary tumors	Required for definitive diagnosis

**Table 2:** Immunohistochemical differential diagnosis of a carcinoma involving the seminal vesicle, including the lineage-specific markers of urothelial and intestinal differentiation.

Tumour	CA-125	CK7	CK20	PSA/PAP	Other
Seminal vesicle adenocarcinoma	+ (most)	+	−	−	PAX8 variable (not defining), MUC6 +, CEA variable; GATA3 −, p63/p40 −, uroplakin II −
Prostatic adenocarcinoma	−	+/−	−	+	NKX3.1 +, AMACR +; GATA3 −, p63/p40 −
Urothelial (bladder) carcinoma	−	+	+/−	−	GATA3 + (~75%; lower in upper tract and in variants), p63/p40 +, uroplakin II + (specific but insensitive), S100P +; in CIS aberrant full-thickness CK20, abnormal p53, CD44 loss
Rectal adenocarcinoma	−	−	+	−	CDX2 +, SATB2 +; GATA3 −

“+” indicates that the marker is present; “–” indicates that it is absent.

**Table 4:** Selected illustrative cases of primary seminal vesicle malignancy spanning the histological spectrum. RT, radiotherapy; MVAC, methotrexate, vinblastine, doxorubicin, cisplatin; SV, seminal vesicle; IHC, immunohistochemistry. This table is the authors’ own compilation, summarising previously reported cases from the cited sources in the authors’ own words; no table, figure, or other copyrighted material from those publications is reproduced.

Study	Age/Sex	Histology	Treatment	Outcome
Ormsby et al. 2000 [15]	4 cases	Papillary adenocarcinoma (IHC series)	—	Defined CA-125+/CK7+/CK20− profile
Yin & Jiang 2018 [12]	51/M	Adenocarcinoma (CA-125 137 U/mL)	Surgery, paclitaxel–cisplatin, RT 60 Gy/30	Disease-free at 5 years
Terrisse et al. 2017 [23]	43/M	Adenocarcinoma, metastatic (lung)	Dose-dense MVAC → surgery → metastasectomy	Complete remission at 48 months
Yao et al. 2023 [18]	54/M	Adenocarcinoma with long-standing SV cyst	Robot-assisted resection, RT 66 Gy/30	No recurrence at follow-up
Alaoui Mhammedi et al. 2023 [13]	25/M	Mucinous adenocarcinoma (cyst-mimic)	Radical excision	Incidental, localised disease
Murakami et al. 2022 [16]	27/M	Clear cell carcinoma (Müllerian)	Laparoscopic radical prostatectomy	Local recurrence at 1 year
Yuan et al. 2024 [24]	43/M	Clear cell adenocarcinoma, ovarian homology	Laparoscopic excision	Well at 20 months
Franco-Buenaventura et al. 2022 [7]	43/M	Primary seminoma of seminal vesicle (2nd reported case)	Radical surgery followed by BEP chemotherapy	No evidence of disease at 1 year
Kreiner et al. 2010 [62]	70/M	Neuroendocrine carcinoma (Lambert–Eaton syndrome)	—	Aggressive; poor prognosis

**Table 5:** Proposed minimum dataset for the reporting of primary seminal vesicle neoplasms. The dataset is intended for authors of case reports and small series and for cancer registries, and requires only information generated in the course of routine surgical and pathological practice. It is not a staging system, and it is not validated; its purpose is to render future reports comparable so that the prognostic questions can eventually be addressed.

Item	What Should Be Recorded
Laterality and centre	Right, left, or bilateral; whether the radiological and pathological centre of the mass lies within the seminal vesicle
Size	Maximum tumour dimension in millimetres, on imaging and on the resection specimen
Local extent	Confined to the seminal vesicle, or invading a named adjacent structure (prostate, bladder, distal ureter, rectum, pelvic side wall), each recorded separately rather than pooled as “locally advanced”
Margin status	R0, R1, or R2, with the site of any involved margin specified
Nodal status	Number of nodes examined and number involved, by named station (obturator, external iliac, internal iliac, common iliac, para-aortic)
Distant disease	Present or absent at diagnosis, with sites named and the method of assessment stated
Histology	Tumour type according to the classification used in this review, with grade where applicable, and whether the lesion is a carcinoma or a non-epithelial or biphasic neoplasm
Immunohistochemistry	The full panel applied and the result of each marker, including negative results
Treatment	Extent of surgery, and the dose, field, and timing of any radiotherapy and the regimen and number of cycles of any systemic therapy
Outcome	Duration of follow-up, site and date of any recurrence, and status at last contact, with a minimum of two years reported wherever possible

Abbreviations: R0, microscopically margin-negative resection; R1, microscopically involved margin; R2, macroscopic residual disease.

## Data Availability

No new data were created or analysed in this study. Data sharing is not applicable to this article.
